# Does eating eggs matter?

**DOI:** 10.20945/2359-3997000000464

**Published:** 2022-04-19

**Authors:** Eder Carlos Rocha Quintão

**Affiliations:** 1 Medicina Interna São Paulo SP Brasil Medicina Interna, Universidade de São Paulo, São Paulo, SP, Brasil

**Keywords:** Eggs, atherosclerosis, cholesterol, diet, meta-analysis

## Abstract

Dietary cholesterol is absorbed in proportion to the amount ingested, blocking its hepatic synthesis, increasing its biliary excretion, only slightly increasing production of bile acids while potentially raising the serum concentration of the atherogenic low-density lipoprotein. Humans lie midway between rats and rabbits that respond to dietary cholesterol, respectively, with high and low capacity to produce bile acids, and low or high capacity to raise blood cholesterol. There are regular studies exonerating as well as blaming dietary cholesterol as a cardiovascular risk factor, particularly in genetic hypercholesterolemic individuals. We then resorted at reviewing all meta-analyses on the subject but failed to reach at a clear conclusion useful in medical practice. Nevertheless, ingestion of the same amount of cholesterol results in wide variation in the amounts absorbed and in plasma lipoprotein profiles depending on poorly understood genetic factors. Several genetic conditions are capable of interfering with the absorption and synthesis of cholesterol. Hyperabsorption of dietary cholesterol elicits the accumulation of cholesterol in the liver and in plasma. In this regard, most cases of familial hypercholesterolemia that have a case of intestinal hyperabsorption of cholesterol also demonstrate the same defect. A practical useful suggestion is to measure for a few weeks the total serum cholesterol and its fractions at least three times before and during the intake of eggs that the candidate wishes to maintain in his usual dietary practice as an efficient procedure to identify those who respond with undesirable increases in serum cholesterol.

## INTRODUCTION

The discovery in 1913 by Nikolay Anitschkow in Russia that dietary cholesterol raises blood cholesterol in rabbits and produces experimental atherosclerosis ( 1 ) led after World War II to population studies initiated in the USA on the role of diet in cardiovascular disease since 1948. There was thus a long interregnum between discovery and public awareness of cholesterol importance ( 2 ).

At the same time, several metabolic studies in humans have shown that dietary cholesterol is absorbed in proportion to the amount ingested, blocking the hepatic synthesis of cholesterol, increasing biliary excretion of cholesterol, but only slightly increasing production of bile acids ( 3 , 4 ). It also raises low-density lipoprotein, which is atherogenic, more than the anti-atherogenic HDL ( 5 ).

It should be added that in all animals investigated cholesterol ingestion raises blood cholesterol to a greater or lesser degree, as in baboon, cat, dog, goat, guinea pig, hamster, several monkeys and birds, mouse, pig, and rat. However, clearly, the responses of cholesterol metabolism differ greatly among the animals mentioned. It is impossible to exclude humans since Carl Müller's in 1939 showed familial hypercholesterolemia as a cause of premature cardiovascular disease ( 6 ). These results have ethically precluded population-based investigations of risk for cardiovascular disease based on egg or cholesterol intake.

Many population-based investigations of the role of dietary cholesterol in risk for cardiovascular disease have since emerged. However, in the Western world, none of these have been carried out promoting cholesterol intake as such or as eggs, the main source represented by the amount ingested, although of less importance than dairy products and red meat in the overall Western diet ( 7 ). Even if its absorption is blocked by foods, such as phytosterols, and drugs, such as ezetimibe, the cost of this type of investigation is prohibitive because it requires a long time and a large number of participants ( 8 ).

In the absence of prospective studies based solely on cholesterol intake, it is not surprising that the results of investigations give rise to questions since cholesterolemia is influenced by numerous biological factors, such as heredity and associated pathologies. In addition, in premature cardiovascular disease, there are adverse modifiable factors – except for the favorable effect of alcohol – such as smoking habit, obesity, sedentary lifestyle, hypertension, diabetes mellitus, genetic causes of hypercholesterolemia, elevations of dietary sodium, trans-fatty acids, and saturated fatty acids ( 9 ). In general, foods high in cholesterol are also high in saturated fatty acids, which seem to be the main contributors to the elevation of blood cholesterol ( 10 , 11 ).

## CONTROVERSIAL RESULTS ON HUMAN POPULATIONS

The results in human studies have been so contradictory that they have influenced the information given to the public by the media for decades. A cursory look at the so widely consulted Google system is enough to witness the most nonsensical information possible, and often totally irresponsible in interpreting the problem, that we deem it inconvenient to dwell on examples. Nevertheless, because of their relevance, there are epidemiological factors to be considered. For example, it is suggested that egg yolk may be a very useful food source despite being high in cholesterol ( 12 , 13 ). This information adds to the reviews that conclude that evidence lacks that dietary cholesterol is relevant as a risk for cardiovascular disease ( 14 – 17 ).

We then examined population studies. As for the cardiovascular risk, there are those who exonerate ( 18 – 28 ) and those that show the participation of dietary cholesterol ( 29 , 30 ), or do so only in part in hypercholesterolemic individuals ( 31 ).

## WHAT META-ANALYSES TELL US

Because of these discrepancies, we evaluated the meta-analyses. Several exempt dietary cholesterol as a risk factor ( 32 – 38 ). However, three of them indicate a higher risk in diabetics ( 39 – 41 ). Other authors blame dietary cholesterol in cardiovascular risk ( 42 – 46 ). In particular, Zhong and cols. ( 46 ) conclude quite convincingly that egg intake represents an increased risk of cardiovascular disease, although the original publications used by them do not report results on cholesterol intake that would allow us to draw a conclusion from each publication they used. Interestingly, the meta-analysis by Dehghan and cols. ( 36 ) exempting eggs as a risk factor is criticized by Schwingshackl ( 47 ) for the following reasons: 1) a single food frequency questionnaire was conducted; 2) an included investigated population had a very heterogeneous socioeconomic background that could have influenced the results, and 3) inclusion of a high proportion of Chinese people accustomed to high carbohydrate diet.

Interestingly, with the exception of three meta-analyses ( 36 , 45 , 46 ) all the others used many common investigations between them, which allows us to conclude that they employed diverse, even antagonistic statistics in interpreting the results of the studies. In short, we did not find the desired consistency of interpretation in several meta-analyses so as to allow a clear useful conclusion.

## WOULD IT THEN BE POSSIBLE TO EXTRACT USEFUL CONDUCT FROM THESE INCONGRUOUS RESULTS?

The message we want to get across is a positive one. Ingesting the same amount of cholesterol can result in great variation in the amounts absorbed ( 3 , 48 – 52 ), and in plasma cholesterol concentrations ( 53 – 55 ). Such results depend on various genetic factors and associated pathologies as demonstrated in extensive literature ( 56 – 61 ). There are even exceptional cases, such as a man who ate 25 eggs a day, but the elevation of his blood cholesterol was very slight due to his unusually high capacity to synthesize bile acids ( 62 ). Others are described as marked elevation of blood cholesterol caused by slight increases in cholesterol intake ( 4 , 63 ), or of marked decreases in blood cholesterol induced by dietary phytosterols that block the intestinal absorption of cholesterol ( 64 , 65 ). We demonstrated in moderately hypercholesterolemic subjects distributed in tertiles of plasma LDL-C that the highest tertile responds best to dietary phytosterol in lowering LDL-C. By this procedure, we highlight the link between primary hypercholesterolemia and higher capacity to absorb dietary cholesterol ( 65 ) discussed below.

## COULD PRIMARY HYPERCHOLESTEROLEMIA ARISE FROM INCREASED INTESTINAL ABSORPTION?

In fact, it has been suggested that there are cases of familial hypercholesterolemia without known genetic defects, or at least without the canonical genetic defects, in which the etiology is postulated to be increased cholesterol absorption, and whose cause is not known ( 59 – 61 ). In this regard, several genetic conditions have been described that are capable of interfering with the absorption and synthesis of cholesterol ( 66 , 67 ). Most cases of familial hypercholesterolemia that have a case of intestinal hyperabsorption of cholesterol also demonstrate the same defect ( 59 ). There is at least one pathology, familial combined hyperlipidemia, defined by cholesterol hyperabsorption independent of body weight ( 68 ).

## HOW MUCH CHOLESTEROL DO WE ABSORB AND WHAT ARE THE CONSEQUENCES?

In metabolic studies in humans in which very high amounts of cholesterol are fed, with less repercussion on the elevation of plasma cholesterol ( 69 – 71 ), there is clear tissue and liver accumulation of cholesterol ( 3 , 70 , 71 ),

Ingestion of the same amount of cholesterol results in wide variation in the amounts absorbed ( 48 , 52 , 71 ) and in plasma lipoprotein profiles ( 53 – 55 ). Such outcomes depend on several poorly understood genetic factors and associated conditions, as shown in extensive literature ( 56 – 61 ).

## CONCLUSION WITH A PRACTICAL MESSAGE

How do we reconcile the incongruent results of population-based research dealing with cardiovascular disease risk with the practical reality exposed above of unpredictability of the plasma lipoprotein response to eggs? We advise that the practical and effective way to avoid the undesirable effects is to undergo individual experimentation, necessarily supervised by a nutritional professional expert. Crucial in this experimentation is to maintain strictly stable body weight while measuring for a few weeks the total serum cholesterol and its fractions at least three times before and during the intake of eggs that the candidate wishes to maintain in his usual dietary practice. I suggest about three weeks of experimentation in each period. If the result obtained is within the desirable standards indicated by the consensus of medical societies in the specialty, which can vary among populations, countries, and clinical conditions, the habit can be maintained for life. Particularly in primary hypercholesterolemia, this experiment is desirable. Properly conducted, it provides efficient results and can be carried out until effective future laboratory methods emerge to identify those genetically susceptible to an undesirable response to egg ingestion.
